# Microaggressions towards People with Mental Illness

**DOI:** 10.1192/j.eurpsy.2024.1267

**Published:** 2024-08-27

**Authors:** C. H. Ayhan, O. Sukut, H. Bilgin, F. Tanhan, K. Aslan

**Affiliations:** ^1^Psychiatric and Mental Health Nursing, Van Yuzuncu Yil University, Van; ^2^Psychiatric and Mental Health Nursing, Istanbul University- Cerrahpasa, Istanbul; ^3^Guidance and Psychological Counseling, Van Yuzuncu Yil University, Van, Türkiye

## Abstract

**Introduction:**

Microaggressions, or subtle expressions of discrimination directed towards individuals because of their membership in marginalized social groups, are the subject of a growing body of literature (Sue, 2010). As a result of growing understanding of politically correct beliefs over time, they’ve been defined as subtler types of discrimination that have replaced formerly overt discrimination. Microaggressions differ from traditional prejudice in that they are frequently perpetrated by well-intentioned people who are oblivious of the negative implications and consequences of their conduct. Microaggressions have been documented in a variety of social groups, including racial/ethnic minorities (Sue et al., 2008; Torres et al., 2010), gender (Swim et al., 2001), sexual orientation (Shelton and Delgado-Romero, 2011), and ability status (Shelton and Delgado-Romero, 2011). Many people with mental illnesses have reported social rejection experiences that are similar to microaggressions, according to research (Cechnicki et al., 2011; Lundberg et al., 2009; Wright et al., 2000; Yanos et al., 2001).

**Objectives:**

Existing measures of stigmatizing attitudes and behaviors may not capture much of the nuance in behavior that people with mental illness report to be particularly upsetting, so we thought it would be important to examine reliability and validity of the mental illness microaggressions scale-perpetrator version (MIMS-P) for measuring microaggression behavior in the general public in Turkey.

**Methods:**

The methodological study will be conducted to establish the validity and reliability of the The mental illness microaggressions scale-perpetrator version (MIMS-P) scale to Turkish Culture and to determine the microaggression levels against individuals with mental illness in the general population. The sample of the study will consist of individuals who are reached through an online questionnaire and who agree to participate in the study. Individuals who have psychiatric disorders will not be included in the study.

**Results:**

Data collection process is still ongoing. Description of studies and the key findings will be presented.

**Conclusions:**

The MIMS-P is designed to aid future study on the frequency of endorsement of microaggressions performed against people with mental illnesses, with the ultimate goal of understanding the mechanisms that lead to these acts.

The development of an extra scale to measure microaggressions from the perspective of people with mental illnesses who encounter them is one of the future research objectives.

With a better knowledge of these viewpoints and how they interact, effective therapies and public policy initiatives for reducing stigma against mental illness can be developed.

**Disclosure of Interest:**

None Declared

